# Body surface posture evaluation: construction, validation and protocol of the SPGAP system (Posture evaluation rotating platform system)

**DOI:** 10.1186/s12891-016-1057-0

**Published:** 2016-05-04

**Authors:** Debora Soccal Schwertner, Raul Oliveira, Giovana Zarpellon Mazo, Fabiane Rosa Gioda, Christian Roberto Kelber, Alessandra Swarowsky

**Affiliations:** Department of Physiotherapy, Santa Catarina State University (UDESC), Pos Graduate Program of Human Kinetics Faculty, University of Lisbon (UL), Pascoal Simone, 358, 88080-350 Florianópolis, Brazil; Pos Graduate Program of Human Kinetics Faculty, University of Lisbon (UL), Lisbon, Portugal; Pos Graduate Program of Human Science Movement of Center of Health Sciences and Sport, Santa Catarina State University (UDESC), Florianópolis, Brazil; IEEE Member, Porto Alegre, Brazil; Pos Graduate Program of Physical Therapy of Center of Health Sciences and Sport, Santa Catarina State University (UDESC), Florianópolis, Brazil

**Keywords:** Quantitative analysis of body posture, Evaluation system, Photogrammetric method

## Abstract

**Background:**

Several posture evaluation devices have been used to detect deviations of the vertebral column. However it has been observed that the instruments present measurement errors related to the equipment, environment or measurement protocol. This study aimed to build, validate, analyze the reliability and describe a measurement protocol for the use of the Posture Evaluation Rotating Platform System (SPGAP, Brazilian abbreviation).

**Methods:**

The posture evaluation system comprises a Posture Evaluation Rotating Platform, video camera, calibration support and measurement software. Two pilot studies were carried out with 102 elderly individuals (average age 69 years old, SD = ±7.3) to establish a protocol for SPGAP, controlling the measurement errors related to the environment, equipment and the person under evaluation. Content validation was completed with input from judges with expertise in posture measurement. The variation coefficient method was used to validate the measurement by the instrument of an object with known dimensions. Finally, reliability was established using repeated measurements of the known object.

**Results:**

Expert content judges gave the system excellent ratings for content validity (mean 9.4 out of 10; SD 1.13). The measurement of an object with known dimensions indicated excellent validity (all measurement errors <1 %) and test-retest reliability. A total of 26 images were needed to stabilize the system. Participants in the pilot studies indicated that they felt comfortable throughout the assessment. The use of only one image can offer measurements that underestimate or overestimate the reality. To verify the images of objects with known dimensions the values for the width and height were, respectively, CV 0.88 (width) and 2.33 (height), SD 0.22 (width) and 0.35 (height), minimum and maximum values 24.83–25.2 (width) and 14.56 – 15.75 (height). In the analysis of different images (similar) of an individual, greater discrepancies were observed in the values found. The cervical index, for example, presented minimum and maximum values of 15.38 and 37.5, a coefficient of variation of 0.29 and a standard deviation of 6.78.

**Conclusions:**

The SPGAP was shown to be a valid and reliable instrument for the quantitative analysis of body posture with applicability and clinical use, since it managed to reduce several measurement errors, amongst which parallax distortion.

## Background

The posture evaluation instruments most cited in the literature are: posture evaluation through observation [1–3], X-ray examination [4, 5], flexible ruler [6–8], photography, film [9–11] and scanner [12, 13].

Visual observation, based on the posture evaluation card is still widely employed due to its low cost, but it only provides qualitative and subjective data of the image under observation, requiring an expert evaluation to detect details and deviations [2, 3, 11]. It consists of the observation of the subject, wearing a minimum of clothing, from the front, back and side views, any deviations being analyzed according to a predetermined guide based on the ideal alignment [14, 15]. For example, in the ideal sagittal alignment, the gravitational line passes through the external acoustic meatus, the bodies of the cervical vertebrae, the tip of the shoulder, the mid-point of the thorax, slightly behind the hip joint, slightly in front of the knee joint and immediately preceding the lateral malleolus [16].

X-ray examinations (XR) are routinely used to measure curvatures of the vertebral column and to analyze vertebral conditions [4]. X-rays have been considered the golden standard regarding the observation of posture deviations [5], despite being an invasive examination in which the individual is exposed to radiation [17]. The radiation used in X-ray equipment has an accumulative effect in the organism, and each new incidence increases the health risk. The negative effects can be seen soon after exposition (erythema, tissue necrosis), or after a long period of latency (6–25 years), even after low exposition, involving chemical damage to the DNA molecules, increased cancer risk and risk of genetic defects [18].

X-ray studies are also limited because they are done without calibration, and errors can be found when comparing the same measurements from different X-ray images [19].

The flexible ruler is a non-invasive instrument that provides a low-cost quantitative evaluation of spinal curvatures in the sagittal plane, and is easy to use and transport. It is 60 cm long, made of plastic-coated lead, and is only flexible in one plane. After molding to the individual’s spine, the mold is transferred to a sheet of paper where the values in millimeters (length and height) of the spinal curvatures are calculated [20]. Studies have shown excellent levels of inter- and intra-evaluator reproducibility and strong correlation between the two methods (flexible ruler and X-rays). Although the relative mean differences between the flexible ruler and radiologic data are small (<1°) for both the thoracic and lumbar curvatures, the range of values is quite wide (±16°), the symmetrical distribution of the values for both curvatures suggesting a random error. In fact, this can be a result of measurement errors, such as differences in the pressure applied to the equipment in contact with the curvature, which can alter the mold and hence the final measurement [7].

The laser acquisition system used in scanners, is one of the most precise devices [12, 13], but is very expensive and requires that the person being measured remains completely still throughout the measurement. Scanners consist of a 3D (three-dimensional) optical measuring system that produces a digital copy of the surface geometry of a human body [21]. The laser is fixed in the equipment and the video cameras move step by step scanning the individual’s body. The triangulation sensors used move in different directions in the horizontal (total of 360°) and vertical (from top to bottom) planes, and once the values of the X and Y directions are known, they are moved one step in the Z direction (depth). The object is thus mapped bit by bit using the information of the distances between the points [22].

Photography or filming methods enable clinicians to detect postural changes with time, and inter-relate various body parts through measurements with specific software. Photography or filming are low cost methods which are easy and quick to use [10], but they require several methodological steps such as the choice of environment, camera position, resolution of the image captured, and the use of anatomical markers [10, 11] to standardize the photos/films and prevent or reduce distortions and measurement errors. Moreover, there are some limitations inherent to the instruments, such as the analytical variations resulting from the choice of an image that does not correspond exactly to the anatomic plane under evaluation [9], which is called parallax. Since photography provides a two-dimensional image, only the part of the body that is in focus has true measurements, while the other parts might have their dimensions distorted by the effect of parallax [14].

Software is currently available to process two-dimensional images, captured simultaneously by several cameras (between 3 and 6), and reconstruct them in 3D for analysis [23]. However this method is more complex, adds cost, and requires calibration, and is not usually available for analysis in surgeries and clinics [10].

Due to the limitations presented by current posture evaluation instruments due to measurement errors, this study aims to build, validate, analyze the reliability of and describe a protocol for use with the Posture Evaluation Rotating Platform System (SPGAP). This device can be used for quantitative and non-invasive body posture evaluations and is easy to transport and handle, which allows for the control of important measurement errors in the clinical practice of posture evaluation.

## Methods

### Construction of the SPGAP posture evaluation system

For the Rotating Platform Posture Evaluation System, the rotating platform, designated as PGA, was first constructed and calibrated using a kinematic system by way of a digital video camera, a PC computer and image processing and analysis software. Using quantitative analysis this system enables one to confront data and compare deviations in the same individual (at different times) and also amongst different individuals, by way of image measurements (frames) captured by the computer.

The PGA rotates the individual under evaluation during the filming procedure, and because of this movement, a sequence of images (*n*) of the individual practically in the same position can be selected, enabling one to obtain the average of the values measured, reducing the parallax obtained from the analysis of a single image.

After elaborating the system, it was validated the reliability and system stabilization verified and a protocol created for its use.

### System description

**Fig. 1:**
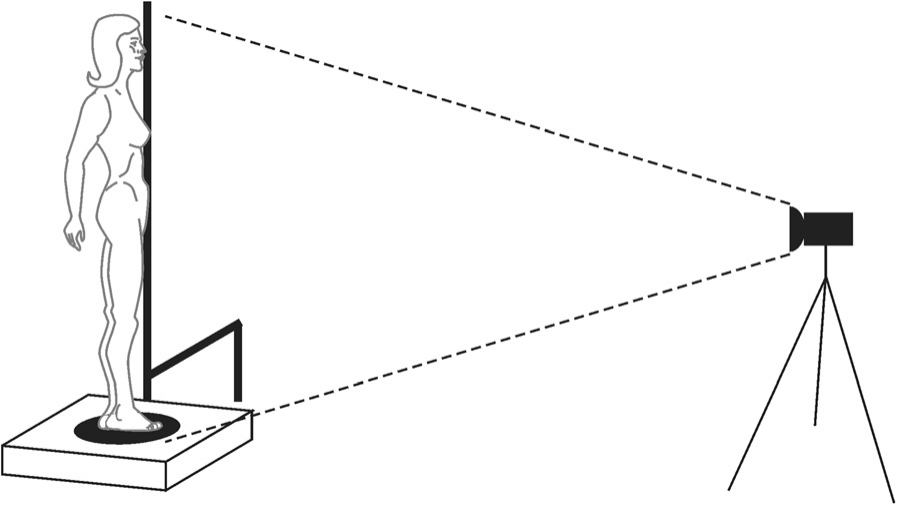
The image acquisition system

The physical components of the system are described in Fig. 1 as follows: the individual positioned on the rotating platform (PGA – Fig. 1 n° 1), the calibration system (Fig. 1 n° 2) close to the platform and in the same plane as the individual under evaluation, and a video camera (Fig. 1 n° 3).

**Fig. 2:**
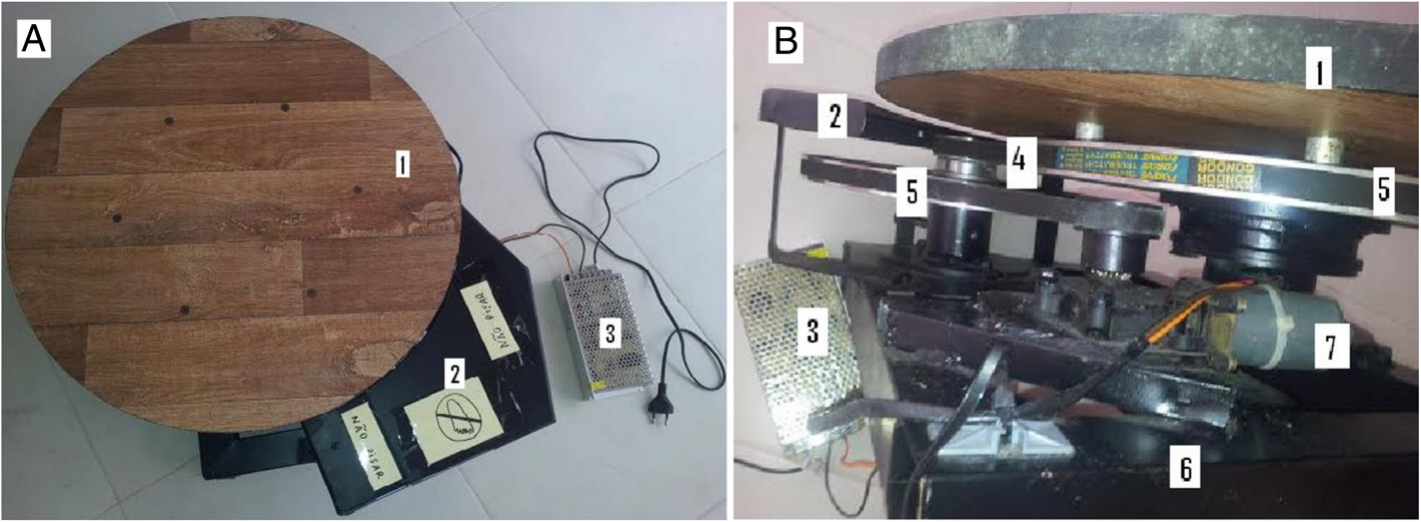
The Posture Evaluation Rotating Platform (PGA) – top view (**a**) and side view (**b**): n° 1 **a** and **b** rotating steel disc, n° 2° **a** and **b** rigid structure covering the pulleys and toothed belt, n° 3 **a** and **b** power supply, n° 4 **b** toothed belt, n° 5 **b** two synchronized pulleys, n° 6 **b** rigid square base, n° 7 **b** DC motor with integrated gearbox

The Posture Evaluation Rotating Platform (Fig. 2a and b) was comprised of a rigid square base (Fig. 2b, n° 6) with 50 cm (centimeter) long sides and a height of 15 cm, and a rotating steel disc with a diameter of at least 35 cm and covered by a rubber material, which was placed in the center (Fig. 2a, b n° 1). In order to start the rotating disc, a mechanical structure was developed (Fig. 2b, n°^s^ 3, 4, 5, 7), which, in addition to allowing for the support of a person of up to 120 kg, served to switch the electrical engine used on and off (Fig. 2b, n° 7).

A single-phase induction engine with a gearbox, either 127 V (Volt) or 200 V, was used on the platform. The coupling between the gearbox axis and the rotating disc was via two synchronized pulleys (Fig. 2b, n° 5) and a toothed belt (Fig. 2b, n° 4), which were under a rigid structure (Fig. 2a and b, n° 2), thus avoiding accidents. As a safety measure, the belt would automatically decouple if an emergency stop occurred. Aiming at the comfort of the individuals to be filmed, the speed of the rotating disc was limited to about 0.7 rpm, which is the equivalent of one turn for every 1.5 min.

The calibration support (Fig. 1 n° 2) had straight segments with distances in centimeters, in order to guide the system with respect to the coordinates and real distances.

A digital camera was used to make the video with suitable resolution (it is important that the camera guarantees the quality and surface recognition). In this study a Sony mini CV 3 mega pixel CCD camera with 30hz of acquisition frequency was used, mounted on a tripod.

**Fig. 3:**
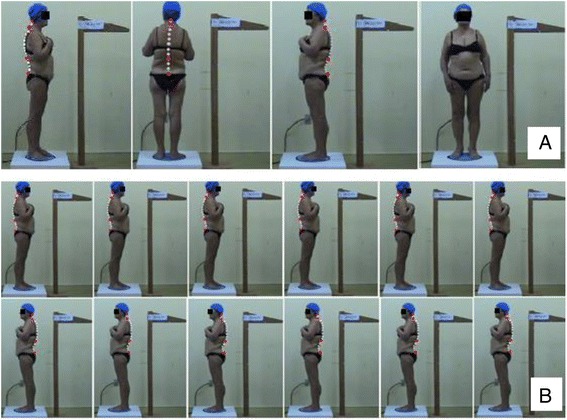
**a** Filming of the individual through 360°, marking of the spinal curvatures (highlight of the limit vertebrae of the curvatures). **b** Model of the frame selections of the person in the sagittal plane

After getting onto the PGA, the individual was rotated through 360° (Fig. 3a) while the video camera was filming. As soon as it was available on the computer, the video file was converted into a set of image files, each containing one frame of the video file (VirtualDub® program or another available one). In order to reduce the parallax, several frames were selected at different degrees of rotation, with the person under evaluation in very similar positions (Fig. 3b), according to the specific interest of the posture evaluation.

**Fig. 4:**
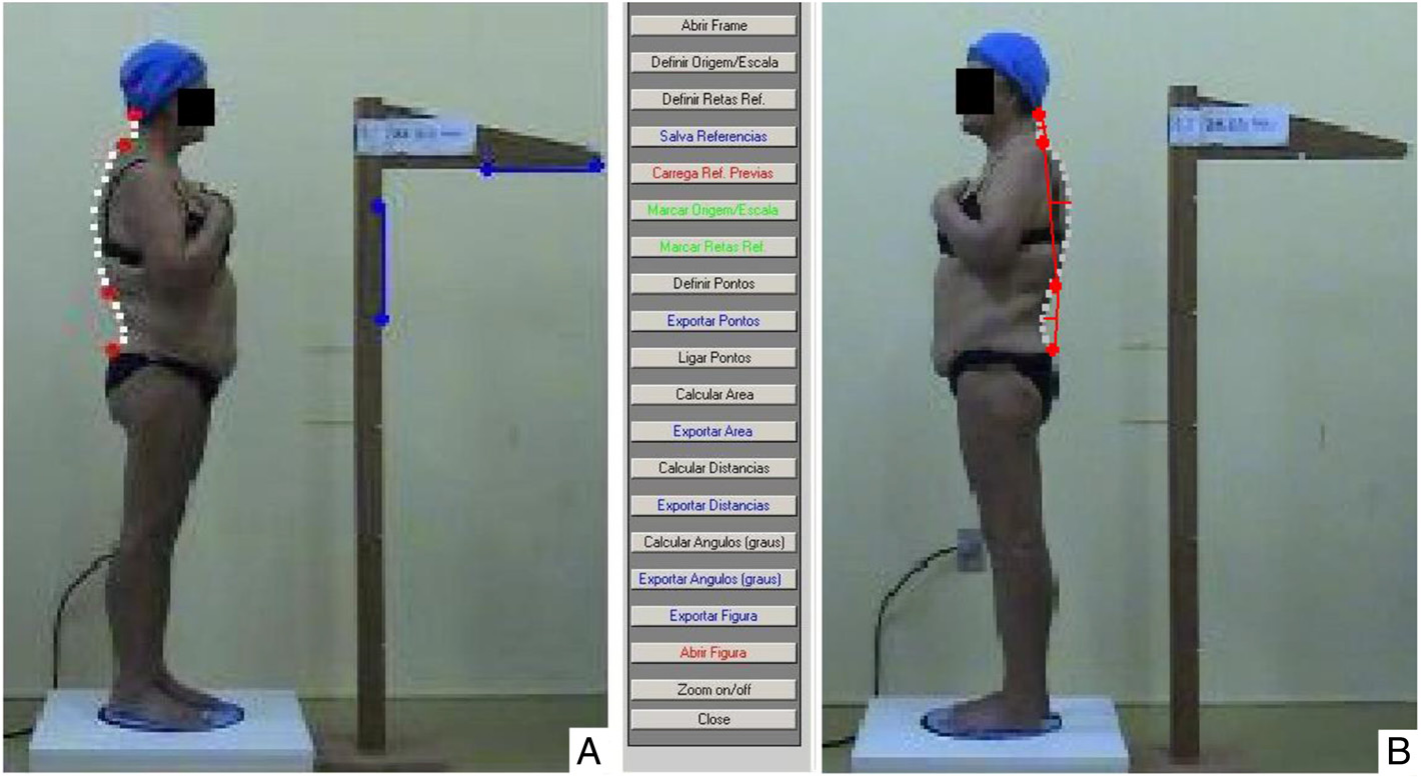
System calibration and measurement **a**) Marks on the calibration support for system calibration (marking of the points on the calibrator with real measurements for the x and y axes); **b** Measurement of the spinal curvatures (used the curvature index)

In order to carry out the other phases of the study, a specific routine was developed to be run using the MatLab® mathematics software, in which after selection of the images, each one was calibrated on the computer, instructing the system regarding the real measurements and coordinates. Figure 4a shows the marks (using the mouse) of the reference points on the calibration support.

From this phase on, the image coordinate axes were defined and adjusted for known coordinates and distances. In this way, any point (pixel) in the image received a coordinate (x,y) related to the defined axes, with distance units in centimeters.

The mouse was then used again to mark the contour surfaces of the curvatures of the cervical, thoracic and lumbar regions, the upper and lower limit vertebrae (Fig. 4b) (based on the marks previously made on the individuals), and any other structure that had to be evaluated. With this information, it was possible to calculate the angles, distances and areas.

**Fig. 5:**
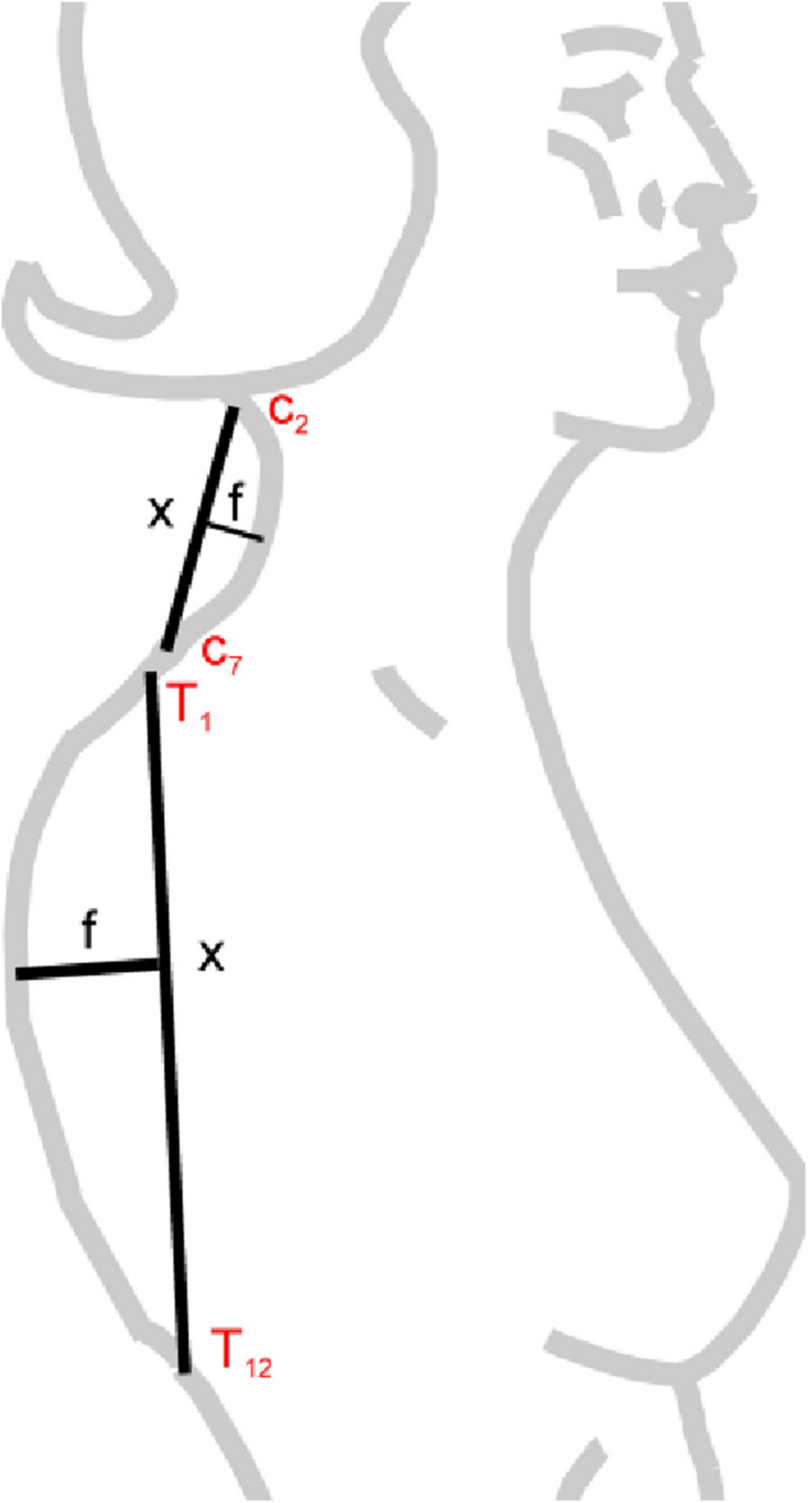
Model for the measurement of spinal curvatures using the curvature index: IC = (f/x) x 100

The curvature index - CI (Fig. 5) was used in this study for this purpose, obtaining the distance between each curve limit vertebra (called straight - x), and the apex of each curve to the straight vertebra (arrow - f) calculated by the formula: CI = (f cm/x cm) x 100. The software automatically calculated all the measurements described.

The values obtained from the processing in all the selected frames were saved in files to be processed later on in the statistical analysis (average value, standard deviation and probabilistic density). In this way, the effects of measurement errors were minimized, mainly those originated from the parallax, in order to obtain the “most accurate” value. It is known that the lower the standard deviation, the lower the influence of measurement errors in the procedure and the closer the results are to the “real values”.

### Posture evaluation system validation, accuracy, reliability and stabilization

The SPGAP system was validated by expert judges using content validation.

The accuracy of the posture evaluation system was verified by comparing the measurements of an object of known dimensions with the values found for the same object using the evaluation system proposed in the present study. The reliability was verified by repetition of the measurement of the same variable (test – retest), and the accumulated coefficient of variation was used to determine the system stabilization, that is, the number of attempts necessary for acceptance of the data measured by the instrument/method.

### Content validation

The content validation aimed at verifying the judges’ opinions about the ability of the SPGAP Posture Evaluation System to analyze posture.

Ten evaluators were selected from different areas of expertise: physical therapy (6), physical education (2), medicine – orthopedics (1) and mathematics (1), in order to verify the system validity, that is, whether, in their opinion, the system measured what it was intended to measure. Information about the study, its objectives and justification, was sent to each evaluator. The evaluators were asked to give a score, from 0 to 10, for each of the following indicators: 1) PGA: a. description of the PGA, b. positioning of the PGA, 2) calibration system: a. description of the calibrator, b. positioning of the calibrator, 3) camera: a. resolution of the vídeo câmera, b. distance between the video camera and the PGA, 4) data acquisition: a. acquisition of the images by the computer, conversion of the film into frames, b. selection of 30 frames to control parallax, and 5) applicability: use of the system to evaluate posture (possibility of calculating distances, angles and areas of body segments). Scores from 0 to 4 meant that the indicator was not valid and would therefore have to be substituted, scores from 5 to 7 indicated it was low and would have to be corrected, and indicated from 8 to 10 indicated it could be considered valid.

### Criterion validity

The accuracy of the posture evaluation system was verified by comparing the measurements of an object of known dimensions with the values found for the same object using the evaluation system proposed in this study. The bidimensional picture of a 25 cm wide by 15 cm high rectangle was used. The object was placed on the rotating platform, 150 cm above the platform base, and filmed while the platform rotated. The computer was employed to calculate the values measured for the rectangle (height and width) in 30 frames of the frontal plane, and all the frames were practically in the same position.

After obtaining the measurements from the 30 frames, the reliability and the variation coefficient analysis were verified. The accumulated average ($\overline{\mathrm{X}}$ accum.), accumulated standard deviation (σ accum.) and accumulated variation coefficient (CV accum) were calculated.

### Determination of the number of repetitions

The number of repetitions necessary to stabilize the system was also calculated, employing the accumulated variation coefficient method.

Verification of the stabilization by calculating the accumulated variation coefficient is amongst the various statistical options used to determine the ideal number of repetitions for an event/test; indicating the number of attempts necessary to accept the data measured by the instrument/method. The variation coefficient is an important measurement concerning the variability of the results, and can be useful in defining the number of repetitions of the assays required. Variability between repetitions can generate an error, and the greater the variability, the greater the variability coefficient, the lower the accuracy and the greater the number of repetitions necessary to represent a determined character [24].

### Pilot study

The protocol to use with SPGAP was established via two pilot studies, which verified factors regarding the environment such as: light (amount of light sufficient to visualize the images, but avoiding reflections), equipment position (distance between the PGA and the video camera, time and difficulty to place the PGA, calibrator and video camera properly levelled), and the individual under evaluation: such as position (standardization of posture in terms of position of the feet, breathing, body position and time for familiarization of the individuals with the PGA), clothing (analysis of the most adequate type of clothing for maximum observation of the posture without embarrassment of the individuals, solutions for unexpected limitations, such as long hair covering the cervical region), marks (need to mark the anatomical points and the difficulty in making the marks and visualizing them in the images captured by the video camera) and sensations throughout the evaluation (search for information related to feelings of dizziness and of feeling unwell caused by the evaluation system).

Each pilot study included 51 elderly individuals (belonging to the Study Group of the Elderly of the Santa Catarina State University/Brazil (UDESC), with a total of 102 evaluations (22 men and 80 women, average age of 69 years old, PD = ±7.3). Each participant signed an Informed Consent Form to take part in the study, which was approved by the UDESC ethics Committee under the registered number of 162/06. The inclusion criteria were: 60 years of age or over (ideal to test the platform, since increase in age provokes greater body unbalance and posture deviation), not presenting mental, cognitive or physical problems that would make it impossible to carry out the interview and posture evaluation (so that the elderly could cooperate by reporting their opinions and body sensations throughout the evaluation), and that they agreed to take part in the study.

### Protocol used

Posture evaluation must be carried out individually in an isolated, well ventilated and well-lit place. The light must be controlled to avoid reflections, and at the same time enable visibility of the marked points during filming.

Marking strategic anatomical points is important to quantify postural deviations, since it enables the evaluator to define the limits and magnitudes of the regions that are to be measured.

The individual under evaluation is instructed to remain standing on the platform looking straight ahead, and maintain a relaxed posture, which provides better stability and balance. In order to enable better posture visualization, the person under evaluation must be wearing adequate clothes for the evaluation (the minimum possible). When the hair hampers observation of the cervical region, wearing a swimming cap is recommended.

The platform (PGA) is first leveled and the camera then installed on a tripod, adjusted and also levelled. Considering the distance between the tripod and the platform, it is important to frame the portion to be evaluated on the video, centralizing it, with the calibration support being visible, placed next to the rotating platform at the same level as the individual under evaluation, without spare space being left on the screen. In addition, the distance from the camera should be the same for all individuals under evaluation, since shorter distances provide better visualization of the portion under evaluation and greater accuracy of the data under analysis. When the participants’ height is measured, the shortest distance between the camera and the PGA should be used, which captures the entire image of the tallest person under evaluation, and this distance should be used for all the participants.

In order to familiarize the individual under evaluation with the equipment, one complete rotation should be done with evaluator assistance and support, and repeated until the individual feels sufficiently at ease to carry out the test without assistance. In the pilot studies carried out with this equipment, all the participants felt sufficiently at ease to start filming before one rotation was completed.

After being familiarized, the individual under evaluation remained in position on the platform, without evaluator assistance, and filming was then started together with rotation of the platform. Recording of the individual through 360° (two complete turns per participant, since some masking or improper movement can occur during filming) is recommended. Throughout recording in the sagittal plane, the individual under evaluation was asked to bend his/her elbows and cross the hands on the chest without changing the posture, making it easier to visualize the contours of the curvatures (the use of large markers might be necessary in this plane in order to see the curvatures better and reduce the overlapping of fat tissue).

When analyzing the video file after data collection, in order to reduce the parallax error, 26 frames of the individual under evaluation practically in the same position, but at different angles, were selected. For example, when evaluating the sagittal curvatures, 26 frames taken from the side, perpendicular to the camera close to a 90° angle, were selected.

## Results

### Validation, accuracy, reliability and stabilization of the posture evaluation system

#### Content validation

The result of the judges’ evaluation of the SPGAP was that the system was valid, obtaining a score of 9.4 (SD 1.13). No corrections or modifications were suggested.

#### Accuracy and reliability determinations (% CV)

The values obtained in the SPGAP for the object were very close to those of the real measures (an average width of 24.99, and an average height of 14.99). In the test retest, the equipment presented an error of about 1 % for the dimension of height and 0.3 % for the width, showing that the system was highly reliable (Table 1).

**Table 1:** Values calculated - $\overline{\mathrm{X}}$ acum., σ acum., CV acum, as from the object with known dimensions (25 cm wide rectangle with a height of 15 cm) using the postural evaluation system with 30 frames

Frames	wide	$\overline{\mathrm{X}}$ accum wide	σ accum wide	CV accum wide	high	$\overline{\mathrm{X}}$ accum high	σ accum high	CV accum high
1	24,75	24,75	0	0	15,41	15,41	0	0
2	24,75	24,75	0	0	15,41	15,41	0	0
3	24,75	24,75	0	0	15,41	15,41	0	0
4	24,95	24,8	0,025	0,1	15,41	15,41	0	0
5	25,12	24,86	0,050	0,2	15,22	15,37	0,017	0,11
6	24,75	24,84	0,052	0,2	14,84	15,28	0,051	0,33
7	24,89	24,85	0,052	0,2	14,94	15,23	0,073	0,46
8	24,92	24,86	0,052	0,2	14,94	15,2	0,089	0,58
9	25,1	24,89	0,055	0,22	14,84	15,16	0,10	0,66
10	25,25	24,92	0,062	0,25	14,94	15,14	0,11	0,73
11	25	24,93	0,066	0,26	15,41	15,16	0,11	0,79
12	25	24,93	0,069	0,28	15,41	15,18	0,11	0,72
13	25	24,94	0,071	0,28	14,75	15,15	0,11	0,79
14	24,75	24,93	0,071	0,28	15,41	15,17	0,11	0,72
15	24,75	24,913	0,070	0,28	14,56	15,13	0,11	0,73
16	25,8	24,973	0,073	0,29	14,56	15,09	0,12	0,79
17	24,75	24,96	0,074	0,3	14,56	15,06	0,12	0,8
18	24,89	24,95	0,074	0,3	15,22	15,07	0,12	0,8
19	24,83	24,95	0,074	0,3	14,66	15,05	0,13	0,86
20	24,93	24,95	0,073	0,29	14,66	15,03	0,13	0,86
21	24,95	24,95	0,073	0,29	14,66	15,013	0,13	0,87
22	25	24,95	0,072	0,29	15,22	15,02	0,14	0,93
23	25	24,95	0,072	0,29	14,66	15	0,14	0,93
24	25	24,95	0,071	0,28	14,56	14,98	0,14	0,93
25	25,2	24,96	0,071	0,28	14,66	14,97	0,14	0,93
26	25,1	24,97	0,071	0,28	14,66	14,96	0,15	1
27	25	24,97	0,071	0,28	14,66	14,95	0,15	1
28	25,12	24,97,	0,071	0,28	15,22	14,96	0,15	1
29	25,14	24,98,	0,071	0,28	15,75	14,99	0,15	1
30	25,2	24,99	0,071	0,28	15,03	14,99	0,15	1
$\overline{\mathrm{X}}$	24,99				14,99			
σ	0,22				0,35			
CV	0,88				2,33			

#### Determination of the number of repetitions

**Fig. 6:**
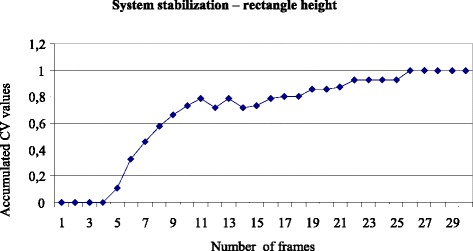
Number of frames necessary to stabilize the system according to the accumulated CV values regarding the rectangle height

**Fig. 7:**
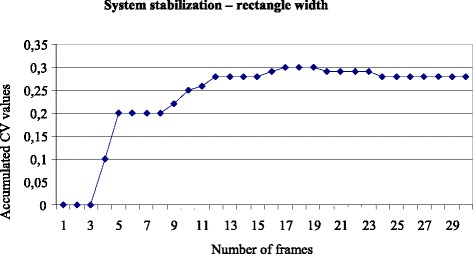
Number of frames necessary to stabilize the system according to the accumulated CV values regarding the rectangle width

Figures 6 and 7 show the number of frames necessary to stabilize the accumulated variation coefficient values.

It can be seen that the number of frames or repetitions (experimental/sample unit size) needed to stabilize the system was 26 repetitions for the height and 18 for the width. Therefore, at least 26 frames were necessary to stabilize the system.

## Discussion

The construction of the SPGAP sought to control measurement errors such as parallax, through the analysis of several images of the participant almost in the same position. The use of a single image could offer measurements that under- or overestimate reality. On verifying the images of objects with known dimensions, the values for width and height showed the following data: CV of 0.88 for width and of 2.33 for height; standard deviation of 0.22 for width and 0.35 for height, minimum and maximum values of 24.83 and 25.2 for width and of 14.56 and 15.75 for height. In the analysis of the different, similar images of an individual, a greater discrepancy in the values was noted. The cervical index showed minimum and maximum values of 15.38 and 37.5, coefficient of variation of 0.29 and standard deviation of 6.78. The thoracic index showed minimum and maximum values of 10 and 24.32, coefficient of variation of 0.28 and standard deviation of 4.52. The lumbar index showed minimum and maximum values of 11.11 and 18.75, coefficient of variation of 0.17 and standard deviation of 2.7.

This difference in the values found could be related to parallax, to evaluator errors, and in the case of the observation of various images of the individual, to changes in the posture of the individual (posture correction, tiredness, movements).

Thus on selecting a single image of the individual, the value found is attributed as real, without considering the other possible values, whereas analyzing various images and attributing a mean value and standard deviation helps to control these errors. It was observed that the greater the postural deviations in the cross-sectional plane (rotations) and the greater the volume of adjacent tissue (skin and fat), the greater the difference in the values obtained during the measurements of the images selected for the same individual.

To help control these errors, one important procedure in the evaluation is the standardization of the instructions given to the individuals under evaluation before the posture evaluation [25], such as feet and arm position and breathing, since standardizing these details enables the comparison of images. The SPGAP used does not require a pre-set feet positioning, since it is understood that the position in which the individual is natural, relaxed, looking straight ahead and with a normal breathing rate is the most suitable one for the evaluation. In agreement with this point of view, Bullock-Saxton [26] pointed out that using the most comfortable standing posture of the individual at the moment of the evaluation can be representative of the real alignment. However, Ferreira et al. [25] used a rubber rug with a drawing of the feet, on which the individual under evaluation should place his feet so that the same position was maintained for all four photographs (sides, front, back).

Apart from the position of the individual under evaluation, some care should be taken with the environment and equipment to guarantee minimum quality for the photogrammetric analysis, such as: lighting, ventilation, camera position, correct marking of the specific anatomic points, system calibration and image resolution [11, 27]. Such care must be present in all protocols and that of the SPGAP closely followed the recommendations found in the literature.

Marking anatomic points is relevant to the quantification of deviations in photogrammetry/kinematics, but it is also a frequent source of measurement errors. According to Saad et al. [28] marking is subject to the interference of soft tissue, and significant alterations might occur in the correspondence of the marker on the skin in relation to the anatomic segment. Furlanetto et al. [29] and Saad et al. [28] also suggested that the marking of points might be compromised when palpation occurs in a different position from that to be evaluated, and hence in order to avoid these errors, marking should be always carried out by the same evaluator and in the position to be evaluated. In this study, the elderly individuals were marked in the same position as that in which they would be evaluated, and all by the same evaluator, who has about 15 years of experience in posture evaluation. Difficulties were found in the palpation of some spinous processes due to the excess of fat tissue and skin. Harlick et al. [30] also verified that manipulative physiotherapists faced some difficulties in locating lumbar spinal processes through palpation of the skin surface. In order to see the contours of the curvatures better in the SPGAP, overcoming the overlap of soft tissue in the image captured, polystyrene balls were fixed to the spinal processes with transparent adhesive tape and the limit vertebrae of each curvature was marked with colored balls (to differentiate them from the other markers). Canales et al. [31] and Ferreira et al. [32] also used polystyrene balls, fixed with transparent adhesive tape onto the vertebrae so as to better visualize the column in the photographs taken from the side.

With respect to positioning of the camera, it should first be placed on the tripod and properly levelled [14]. The PGA and calibrator should also be properly levelled. The distance of the camera was calculated to provide adequate resolution with less dead space, since dead space reduces system resolution by decreasing the quantity of pixels available to represent the capture volume. Sacco et al. [14] identified the best image size and resolution to analyze body posture, that is, the minimal pixel density required to identify the anatomical markers and body segments on the monitor, and guarantee the reliability of the angular and linear measurements in the postural evaluation by kinematics/photogrammetry. They concluded that an image with 768 pixels analyzed on a 96 ppi screen could provide a very good image. In this study they opted for the use of a CCD 3 megapixels (2048x1536 pixels) video camera, which can be acquired for an accessible price.

Regarding verification of the accuracy of SPGAP, an object with known dimensions was used and the real measurements compared with the values found for the same object using the evaluation system. This information helped reduce the measurement error related to the latent variables of the individuals when under evaluation (e.g. posture variation, movements, tiredness). This latent error was confirmed by Tomkinson, Grant and Shaw [14] who evaluated asymptomatic adults and shop dummies using the scanner, and found that most of the measurement errors were related to the posture error and not a technical error. Brink et al. [33] also used dummies in their study to verify the reliability of the instrument, in an attempt to eliminate measurement errors related to the variability of the individual.

## Conclusions

The posture evaluation system developed in this study presented a simple and practical protocol for the quick analysis of posture. In addition the system is lightweight and easy to transport and assemble.

The Posture Evaluation Rotating Platform was shown to be valid, reliable and a suitable equipment for the quantitative analysis of body posture with clinical applicability. Measurement errors common to videography such as parallax distortion, which is disregarded in many studies, were reduced.

### Ethics and consent to participate

This study was approved by the UDESC ethics Committee under the registered number of 162/06.

Elderly (over 60 years) who participated in this study received information about the study objectives and the form of participation (data collection procedure, risks, confidential information). Each participant signed an Informed Consent Form and Consent Form for Filming to take part in the study.

The subject appearing in the Figures provided her consent for her image to appear in the paper.

### Availability of data and materials

All data that supports our findings are contained in the manuscript.

## Abbreviations

SPGAP: Posture evaluation rotating platform system (Brazilian abbreviation)
PGA: Rotating platform
$$ \overline{\mathrm{X}} $$: Mean
SD or σ: Standard deviation
CV: Coefficient of variation
XR: X-ray
UDESC: Santa Catarina State University
CI: Curvature index
3D: Three-dimensional
V: Volt
CCD: Charge-coupled device
cm: Centimeter
Accum: Accumulated
hz: Hertz
